# Navigating the Complexity: A Comprehensive Review of Managing Pregnancy in Complete Heart Block Cases

**DOI:** 10.7759/cureus.50977

**Published:** 2023-12-23

**Authors:** Akash Agarwal, Siddhant P Murkey, Pranam Pandit, Arpita Jaiswal, Suyash Agrawal

**Affiliations:** 1 Surgery, Jawaharlal Nehru Medical College, Wardha, IND; 2 Medicine, Jawaharlal Nehru Medical College, Wardha, IND; 3 Obstetrics and Gynecology, Jawaharlal Nehru Medical College, Wardha, IND

**Keywords:** pacing technology, arrhythmias, multidisciplinary care, obstetric management, pregnancy, complete heart block

## Abstract

This review explores the comprehensive management of pregnancy in cases of complete heart block, a cardiac condition characterized by the dissociation between atrial and ventricular conduction. The review begins with a thorough examination of preconception counseling, emphasizing the assessment of maternal health, the evaluation of cardiac function, and the identification of potential risks. Subsequently, the medical management section delves into using beta-blockers, pacemakers, and antiarrhythmic drugs to monitor cardiac function during pregnancy and adjust medication regimens. Obstetric considerations highlight the importance of antenatal care, fetal monitoring, and thoughtful delivery planning, including the choice between vaginal delivery and cesarean section. The section on complications underscores the risks of arrhythmias, heart failure, premature birth, and neonatal cardiac issues. Looking ahead, the future directions and research section explores ongoing studies in genetics, pharmacology, and technological innovations, envisioning potential advancements in pacing technology and personalized medicine approaches. The conclusion synthesizes key findings, offering recommendations for clinical practice and reflecting on the challenges and opportunities inherent in managing pregnancy in complete heart block cases. The multidisciplinary approach emerges as paramount, with collaborative efforts paving the way for improved patient outcomes and advancements in the field.

## Introduction and background

Complete heart block (CHB) (third-degree atrioventricular (AV) block) is a cardiac conduction disorder characterized by complete dissociation between the atria and ventricles. In this condition, the electrical signals generated in the atria fail to reach the ventricles, leading to an independent rhythm between the two chambers of the heart. This disruption in the normal conduction pathway poses a significant challenge to maintaining practical cardiac function [1].

The management of pregnancy in women with CHB is of paramount importance due to the unique physiological demands and potential complications associated with both the cardiac condition and the gestational process. Pregnancy places increased stress on the cardiovascular system. For individuals with CHB, the altered conduction system further complicates the delicate balance required for optimal maternal and fetal well-being [2].

Understanding the intricacies of managing pregnancy in CHB cases is crucial not only for the well-being of the mother but also for the successful progression of the pregnancy and the health of the developing fetus. The potential risks associated with this cardiac condition necessitate a specialized approach to prenatal care, delivery planning, and postpartum management [3].

The purpose of this comprehensive review is to provide a thorough examination of the complexities involved in managing pregnancy in cases of CHB. By exploring the anatomical and physiological aspects of the heart, discussing preconception counseling, and delving into the medical and obstetric considerations, this review aims to offer a comprehensive understanding of the challenges and strategies associated with this unique population.

## Review

Preconception counseling

Assessment of Maternal Health

Preconception counseling plays a pivotal role in optimizing maternal and fetal outcomes in pregnancies complicated by CHB. Assessing maternal health before conception involves a thorough evaluation of the woman's overall well-being and the identification of any pre-existing conditions that may impact the course of pregnancy. This assessment includes a comprehensive review of the patient's medical history, focusing on cardiac and non-cardiac factors that could influence pregnancy [4]. Key components of the assessment process include the review of previous pregnancies, family medical history, and a detailed exploration of lifestyle factors such as smoking, alcohol consumption, and medication use. Identifying and addressing any pre-existing medical conditions, such as hypertension or diabetes, is crucial in optimizing the overall health of the mother before embarking on the challenges of pregnancy in the context of CHB [5].

Evaluation of cardiac function

The evaluation of cardiac function is a critical aspect of preconception counseling for individuals with CHB. Cardiac assessments aim to determine the baseline cardiovascular status of the woman and identify any potential complications that may arise during pregnancy. This involves a combination of non-invasive diagnostic tools, such as echocardiography, electrocardiography (ECG), and ambulatory monitoring, to assess the heart's structural and functional aspects [6]. Echocardiography provides valuable insights into cardiac anatomy, ventricular function, and the presence of any congenital anomalies or valvular disorders. ECG aids in identifying the specific degree of heart block and understanding the electrical activity of the heart. Ambulatory monitoring allows for the continuous assessment of cardiac rhythm over an extended period, aiding in detecting any arrhythmias or fluctuations in heart rate [7].

Identification of Potential Risks and Complications

Once maternal health and cardiac function are assessed, the next crucial step in preconception counseling is the identification of potential risks and complications associated with CHB during pregnancy. This involves carefully considering the physiological changes in pregnancy and their potential impact on the cardiac conduction system [8]. Risks may include an increased incidence of arrhythmias, heart failure, and the potential for adverse outcomes in both the mother and the developing fetus. Understanding the interplay between cardiac conditions and pregnancy-related changes allows healthcare providers to tailor management strategies to mitigate risks and optimize outcomes [9].

Medical management

Medications for Heart Block

Beta-blockers: Beta-blockers play a crucial role in managing CHB by mitigating the effects of sympathetic stimulation on the heart. These medications help control heart rate, reduce arrhythmias, and alleviate symptoms associated with the condition. However, careful consideration is needed, as beta-blockers may affect fetal development. The choice of beta-blocker, dosage, and monitoring parameters should be tailored to the individual patient's needs and the specific characteristics of their heart block [10].

Pacemakers: Pacemakers are often a cornerstone in treating CHB, ensuring appropriate AV conduction by providing artificial pacing when the intrinsic conduction system fails. Preconception counseling should include a discussion of the patient's existing pacemaker, if applicable, addressing issues such as battery life, programming, and the need for potential adjustments during pregnancy. Additionally, considerations for the type of pacemaker and its compatibility with pregnancy-related changes in cardiac demand are essential components of the medical management plan [11].

Antiarrhythmic drugs: In some instances, antiarrhythmic drugs may be prescribed to stabilize cardiac rhythm and prevent arrhythmias. However, the use of these drugs during pregnancy requires careful consideration due to potential fetal risks. The selection of antiarrhythmic medications should be based on a thorough assessment of both maternal and fetal well-being, with close monitoring of drug levels and potential side effects [12].

Monitoring Cardiac Function During Pregnancy

Monitoring cardiac function throughout pregnancy is essential to detect and address any changes or complications promptly. Regular follow-up appointments with a multidisciplinary healthcare team, including obstetricians and cardiologists, are crucial for assessing maternal well-being and fetal development. Diagnostic tools such as echocardiography, ECG, and ambulatory monitoring remain instrumental in tracking cardiac function changes and identifying emerging issues [13]. Particular attention should be given to monitoring heart rate, rhythm, and signs of heart failure. Regular fetal monitoring, including ultrasound examinations and non-stress tests (NSTs), contributes to assessing fetal well-being and detecting any signs of distress related to maternal cardiac function [14].

Adjustments to Medication Regimens During Pregnancy

Pregnancy induces physiological changes that may impact the pharmacokinetics of medications, necessitating careful consideration and potential adjustments to the medication regimen. This includes evaluating the dosage of beta-blockers or antiarrhythmic drugs based on changes in cardiac demand and potential alterations in drug metabolism during pregnancy [15]. Collaboration between the patient's healthcare providers is crucial to balance the therapeutic effects of medications with potential risks to both the mother and the developing fetus. Regular communication and monitoring allow for timely adjustments to medication regimens, ensuring optimal control of the cardiac condition while minimizing potential adverse effects on pregnancy [16].

Obstetric considerations

Antenatal Care

Antenatal care for individuals with CHB involves a comprehensive and multidisciplinary approach to ensure the well-being of both the mother and the developing fetus. Regular prenatal check-ups are essential to monitor maternal cardiac function, assess fetal growth, and address emerging concerns. Antenatal care should be coordinated between obstetricians and cardiologists, optimizing maternal health throughout the pregnancy [17]. Monitoring maternal vital signs, including blood pressure and heart rate, becomes particularly important during antenatal visits. Additionally, routine laboratory assessments and echocardiography contribute to evaluating the impact of pregnancy on cardiac function and guiding any necessary adjustments to the management plan [18].

Fetal Monitoring

Fetal monitoring is a crucial component of managing pregnancies complicated by CHB. Regular ultrasound examinations are employed to assess fetal growth, development, and the presence of any structural abnormalities. NSTs may also be utilized to evaluate fetal well-being by assessing the fetal heart rate in response to movement [19]. Continuous collaboration between obstetricians and cardiologists is imperative to interpret fetal monitoring results in the context of maternal cardiac status. Any deviations from the expected patterns may prompt further investigation and intervention to ensure the optimal health of both the mother and the fetus [20].

Delivery Planning

Vaginal delivery vs. cesarean section: The mode of delivery is a critical consideration in pregnancies complicated by CHB. The decision between vaginal delivery and cesarean section depends on various factors, including maternal cardiac status, the presence of other obstetric complications, and the degree of heart block. At the same time, vaginal delivery is often the preferred mode when there are no contraindications; a cesarean section may be recommended in cases of high-degree heart block or other cardiovascular concerns to minimize the duration of stress on the maternal cardiovascular system [21].

Timing of delivery: Delivery timing is a nuanced aspect that requires careful consideration. Delivering at term is generally the goal, but individualized decisions are based on maternal and fetal well-being. In some cases, early delivery may be indicated if there are concerns about maternal cardiac decompensation or fetal compromise. The delivery timing should be determined collaboratively by the obstetric and cardiology teams, considering the specific circumstances of each case [22].

Multidisciplinary approach

Collaboration Between Obstetricians, Cardiologists, and Neonatologists

The effective management of pregnancies complicated by CHB necessitates a collaborative and multidisciplinary approach. Close coordination between obstetricians, cardiologists, and neonatologists is crucial to address the unique challenges posed by this cardiac condition and to optimize outcomes for both the mother and the newborn [23]. Collaboration begins at the preconception stage, where healthcare providers from different specialties work together to assess the maternal cardiac status, plan appropriate interventions, and establish a comprehensive care plan. Regular communication and joint decision-making ensure that maternal and fetal aspects are considered throughout the pregnancy [24]. During antenatal care, obstetricians and cardiologists collaborate to monitor maternal cardiac function, assess fetal well-being, and make real-time adjustments to the management plan based on evolving clinical needs. The integration of neonatologists into the team becomes increasingly important as the pregnancy progresses, with a focus on preparing for potential neonatal complications associated with maternal heart block [25].

Importance of a Specialized Team in Managing CHB Cases During Pregnancy

Expertise in obstetrics: Obstetricians, as integral members of the multidisciplinary team, bring a wealth of expertise in maternal-fetal medicine to the forefront of managing pregnancies complicated by CHB. Their role extends from the early stages of preconception counseling to the delivery room, focusing on the comprehensive monitoring of maternal health and the assessment of fetal development. Obstetricians are adept at navigating the unique challenges of a CHB, ensuring that the care plan is tailored to each patient's specific needs and conditions. Their expertise becomes particularly crucial in planning a safe delivery, considering the cardiac considerations and potential risks associated with a CHB [26].

Cardiologists' specialized knowledge: Cardiologists contribute specialized knowledge in cardiac conditions, playing a pivotal role in the multidisciplinary team. Their expertise is essential for assessing and managing the intricate impact of a CHB on maternal cardiac function. Cardiologists bring a nuanced understanding of arrhythmias, heart failure, and the dynamic changes in cardiac demand during pregnancy. Their role extends to prescribing and adjusting medications to optimize maternal health while safeguarding fetal well-being. Collaboration with obstetricians is fundamental in determining the optimal mode and timing of delivery, considering both cardiac considerations and obstetric factors. The cardiologist's specialized knowledge ensures a comprehensive approach to managing the cardiovascular aspects of pregnancies affected by CHB [27].

Neonatologists' involvement: Neonatologists contribute critical expertise in managing potential complications that may arise in the newborn due to maternal heart block. Their involvement spans from prenatally preparing for potential issues to addressing any neonatal cardiac challenges postnatally. Neonatologists are vital in caring for newborns, offering specialized interventions and treatments as needed. This includes assessing and managing neonatal cardiac issues associated with maternal heart block and ensuring that the newborn receives prompt and effective care. Their expertise adds a layer of comprehensive support to the multidisciplinary team, addressing the continuum of care from pregnancy to the neonatal period [28].

Case studies and clinical experiences

Presentation of Real-Life Cases

Pregnancy with CHB: A 26-year-old primigravida with CHB at term pregnancy was asymptomatic throughout her pregnancy, with a pulse rate between 50 and 60 beats per minute. The patient can be managed conservatively or may require temporary or permanent pacemaker implantation. Physiological changes in the cardiovascular system during pregnancy may lead to decompensation, particularly during the intrapartum and postpartum [29]. The incidence of complete AV block (CAVB) is estimated to be one in 15,000 to 20,000 live births, and it can be congenital or acquired. The acquired variety is rare during pregnancy and is mainly observed in individuals over 50. In 30% of patients with congenital heart block, the first symptoms occur during pregnancy, likely due to the hyperdynamic circulation of pregnancy. The best route of delivery for such patients is still under debate, and there is no absolute contraindication regarding vaginal delivery [30].

Total AV block in pregnancy (TAVB): TAVB in pregnancy is a rare occurrence that requires a concerted effort involving obstetricians, cardiologists, and intensivists. Pacemaker implantation is recommended, and while vaginal delivery remains the first choice, a cesarean section is indicated under obstetric indications [31].

Management of CHB in a pregnant woman with systemic lupus erythematosus-associated complications: A retrospective clinical case evaluation and review of relevant literature proposed a presumptive association between CHB and the primary diagnosis. The natural history and current therapy for CHB in children and patients with congenital heart disease were also reviewed [32]. These case reports and clinical experiences highlight the challenges and complexities involved in managing pregnancy in cases of CHB, emphasizing the need for a tailored approach and a multidisciplinary team to ensure the best possible outcomes for both the mother and the baby.

*Outcomes and Lessons Learned* 

Managing pregnancy in cases of CHB is complex. It requires a tailored approach based on the individual's condition, involving a multidisciplinary team to ensure the best possible outcomes for the mother and the baby.

Pregnancy with CHB: A case report highlighted the importance of cardiac evaluation in pregnant patients, especially those with a history of syncope. It also emphasized the need for adequate and urgent management in symptomatic cases of CHB in pregnancy and adequate evaluation to decide when to implant the pacemaker as a definitive measure [30]. The best route of delivery for patients with congenital heart block is still under debate, and there is no absolute contraindication regarding vaginal delivery [30].

Total AV block in pregnancy: TAVB requires a concerted effort involving obstetricians, cardiologists, and intensivists. Pacemaker implantation is recommended, and while vaginal delivery remains the first choice, a cesarean section is indicated under obstetric indications [31].

Management of CHB in a pregnant woman with systemic lupus erythematosus-associated complications: A retrospective clinical case evaluation and review of relevant literature proposed a presumptive association between CHB and the primary diagnosis. The natural history and current therapy for CHB in children and patients with congenital heart disease were also reviewed [32].

Predicting postpartum cardiac events in pregnant women with complete AV block: A study investigated predictive factors for postpartum cardiac events in pregnant women with CAVB. The study found that a family history of CHB and perinatal ventricular pause were predictors of postpartum cardiac events [33]. These case reports and studies highlight the importance of early diagnosis, risk stratification, and thorough planning to manage CHB in pregnancy successfully. They also emphasize the need for a multidisciplinary team approach and tailored management based on the individual's condition. While there is no absolute contraindication regarding vaginal delivery, the best route of delivery for patients with CHB is still under debate. Pacemaker implantation is recommended for symptomatic cases of CHB in pregnancy, and temporary pacing during labor may be necessary for some patients.

Variability in Management Approaches Based on Individual Cases

Effectively managing pregnancy in individuals with CHB demands a personalized strategy that considers the unique circumstances of each case. A collaborative effort from a multidisciplinary team is essential to optimize outcomes for both the mother and the baby. The management of pregnant women facing CHB encompasses a spectrum ranging from conservative measures to the consideration of temporary or permanent pacemaker implantation, contingent upon various influencing factors [29]. These factors include the following:

Symptoms and severity of CHB: The spectrum of symptoms associated with CHB, such as syncope, dizziness, and shortness of breath, is pivotal in determining the management approach. The presence and severity of symptoms guide the decision-making process, with symptomatic patients often requiring immediate intervention through temporary or permanent pacemaker implantation. Conversely, asymptomatic individuals may undergo a more conservative management strategy, with regular monitoring and tailored interventions only if symptoms manifest [32].

Type of CHB: The origin of CHB, whether congenital or acquired, significantly influences the management strategy. In cases of acquired heart block during pregnancy, which is comparatively rare, the etiology becomes diverse and may include myocarditis, collagen vascular diseases (e.g., systemic lupus erythematosus), infective endocarditis, or complications arising from prior cardiac surgeries. Understanding the underlying cause is crucial for formulating an effective and targeted management plan [29].

Presence of structural heart disease: The coexistence of structural heart disease is a crucial factor impacting the tolerance of CHB during pregnancy. Patients with underlying structural issues may necessitate a more assertive management approach, potentially involving the implantation of a pacemaker. Structural abnormalities introduce additional considerations, requiring a comprehensive evaluation of maternal and fetal well-being throughout pregnancy [34].

Gestational age: The fetus's gestational age is a critical consideration influencing the timing and intervention approach. Pacemaker implantation during the first trimester poses an elevated risk of fetal loss, necessitating careful deliberation on the optimal timing. Conversely, later implantation during the second or third trimester is associated with an increased risk of preterm labor. Striking a delicate balance between maternal cardiac needs and fetal well-being becomes paramount in decision-making [35].

Delivery plan: The chosen delivery plan further shapes the overall management approach in cases of CHB. Some patients may require temporary pacing during labor, introducing considerations for the optimal delivery route. The debate surrounding the most suitable mode of delivery for individuals with CHB remains ongoing, with the multidisciplinary team weighing the risks and benefits associated with both vaginal delivery and cesarean section based on individualized patient factors [36].

Complications and challenges

Maternal Complications

Arrhythmias: Arrhythmias represent a primary concern in pregnancies complicated by a CHB, given the altered AV conduction system. The inherent risk of bradycardia or other arrhythmias poses potential symptoms such as dizziness, fatigue, or syncope, underscoring the need for vigilant monitoring. Cardiologists play a central role in this aspect, conducting close and regular assessments to detect arrhythmias promptly. Timely intervention is crucial, and adjustments to medication regimens may be implemented to stabilize cardiac rhythm. In severe cases, consideration of pacemaker reprogramming becomes essential to address arrhythmias and ensure optimal maternal cardiovascular function throughout the pregnancy [37].

Heart failure: Maternal heart failure emerges as a significant and potential complication in pregnancies affected by a CHB, mainly due to the heightened physiological demands of gestation. The risk of inefficient cardiac output stemming from impaired AV conduction underscores the importance of careful management. Maintaining fluid balance is critical, as excessive fluid retention can exacerbate heart failure. Medication adjustments, including diuretics and other heart failure medications, may be necessary to optimize cardiac function. Regular cardiac assessments, including echocardiography, are integral to the management strategy, allowing for the early detection and proactive management of heart failure to safeguard the well-being of the mother and the developing fetus [38].

Fetal and Neonatal Complications

Premature birth: The heightened risk of premature birth is a significant concern in pregnancies complicated by CHB. The physiological stress imposed on the maternal cardiovascular system may act as a trigger for preterm labor, necessitating heightened vigilance and timely intervention. Carefully monitoring maternal health, including cardiac function and overall well-being, becomes imperative to detect early signs of preterm labor. In the event of preterm birth, neonates may face challenges associated with prematurity. These challenges necessitate specialized care within a Neonatal Intensive Care Unit (NICU), where comprehensive support is provided to address potential respiratory distress, developmental concerns, and other complications commonly associated with infants born preterm [39].

Neonatal cardiac issues: Neonates born to mothers with CHB may encounter specific cardiac challenges that require thorough consideration. Although the incidence of congenital heart block in newborns is relatively rare, it is a crucial aspect of neonatal care in these cases. Neonatal cardiac issues can range from mild conduction abnormalities to, in more severe cases, CHB. Neonatologists play a central role in monitoring and managing these cardiac complications, conducting regular assessments to identify abnormalities promptly. The potential need for pacemaker placement in the newborn, although not common, underscores the importance of a proactive and multidisciplinary approach to address and mitigate any cardiac issues that may arise in the early stages of the neonate's life. Swift identification and intervention are critical components in ensuring the optimal cardiac health of infants born to mothers with CHB [40].

Future directions and research

Ongoing Research in the Field

Genetic and molecular studies: Ongoing research is at the forefront of delving into the genetic and molecular underpinnings of CHB, aiming to unravel its complex etiology and identify potential genetic markers. Exploring the genetic basis holds promise for a deeper understanding of the condition, paving the way for more targeted and personalized interventions. Advances in genomics may offer the capability to identify individuals at a higher risk of developing CHB, allowing for early interventions and preventive measures. This research avenue represents a crucial step toward unlocking the mysteries of the condition and improving risk stratification in affected populations [41].

Pharmacological interventions: The research landscape continues to evolve in pharmacological interventions for managing CHB during pregnancy. This involves exploring and developing novel medications designed to effectively control arrhythmias while minimizing potential adverse effects on maternal and fetal health. Investigating the safety and efficacy of emerging drugs specific to the pregnant population is an active and dynamic area of study. The ultimate goal is to enhance the pharmacological armamentarium available to healthcare providers, offering more tailored and effective treatment options for pregnant individuals with CHB [42].

Technological innovations in monitoring: Advancements are ushering in a new era for assessing maternal cardiac function and fetal well-being in real time. Wearable devices, continuous remote monitoring, and artificial intelligence applications are poised to revolutionize the healthcare landscape for pregnant individuals with CHBs. These innovations offer the potential for more comprehensive and proactive tracking of changes in cardiovascular status. The integration of wearable devices allows for continuous monitoring outside traditional healthcare settings, providing a more holistic understanding of the dynamic cardiac changes during pregnancy. Such technological innovations hold the promise of enhancing the precision and timeliness of interventions, contributing to improved outcomes and personalized care for individuals navigating pregnancy with CHB [43].

Potential Advancements in Managing CHB During Pregnancy

Precision medicine approaches: The future of managing CHB during pregnancy may witness significant advancements through precision medicine. This approach aims to tailor treatment strategies based on the unique characteristics of each patient, ushering in a new era of personalized care. Precision medicine considers factors such as genetic predisposition and individual responses to medications, allowing for more targeted, effective, and safer management of CHB during pregnancy. Integrating personalized approaches can optimize therapeutic outcomes by considering each patient's genetic makeup and physiological nuances, thus paving the way for a more nuanced and individualized standard of care [44].

Innovations in pacing technology: Ongoing research is dedicated to exploring innovations in pacing technology, specifically focusing on enhancing functionality and adaptability for pacemakers designed for pregnant individuals with CHB. This includes advancements to improve device longevity, optimize battery technology, and incorporate adaptive pacing algorithms. The dynamic changes in cardiac demand during pregnancy necessitate pacemakers that can adapt and respond to these fluctuations. Innovations in pacing technology are poised to provide more robust and flexible solutions, ensuring that pacemakers can effectively meet the unique cardiovascular needs of pregnant individuals, ultimately contributing to improved maternal and fetal outcomes [45].

Fetal intervention strategies: Research endeavors may delve into fetal intervention strategies designed to minimize the impact of maternal CHB on the developing fetus. Innovative approaches, such as in-utero pacing or other interventions, could be explored to optimize fetal cardiac function and reduce the risk of neonatal complications. This frontier represents a pioneering study area with the potential to intervene at the fetal level, aiming to mitigate the consequences of maternal heart block before birth. Such strategies hold promises for enhancing the overall well-being of both the mother and the newborn, addressing potential cardiac challenges in the early stages of fetal development [46].

Long-term follow-up studies: Long-term follow-up studies are essential in advancing the understanding and care of pregnancies complicated by CHB. These studies extend beyond the gestational period, providing insights into the postpartum period and subsequent years. Assessing the long-term effects on maternal cardiac health and understanding the potential impact on offspring offer valuable data for ongoing improvements in care. Longitudinal studies contribute to refining management protocols, enhancing preventive measures, and fostering a comprehensive understanding of the implications of CHB during pregnancy on both the mother and the child's short-term and long-term health outcomes [47].

## Conclusions

In conclusion, the intricate landscape of managing pregnancy in cases of CHB underscores the necessity for a holistic and multidisciplinary approach. The comprehensive review has elucidated key findings, emphasizing the significance of preconception assessments, individualized medical management, and meticulous obstetric considerations. The challenges posed by potential complications, including arrhythmias, heart failure, premature birth, and neonatal cardiac issues, underscore the need for a vigilant and collaborative healthcare team. Recommendations for clinical practice advocate for personalized treatment plans, continuous monitoring, patient education, and robust emotional support. The future of managing CHB during pregnancy holds promise, with ongoing research in genetics, pharmacology, and innovative technologies offering opportunities for refined care. Despite the challenges, the commitment to a patient-centered approach and the collaborative efforts of obstetricians, cardiologists, and neonatologists positions the healthcare community to navigate complexities and foster continuous improvements in care, ensuring the best possible outcomes for mothers and newborns facing this intricate medical scenario.
